# Discovery of a Novel Mutation in DNA Gyrase and Changes in the Fluoroquinolone Resistance of *Helicobacter pylori* over a 14-Year Period: A Single Center Study in Korea

**DOI:** 10.3390/antibiotics9060287

**Published:** 2020-05-27

**Authors:** Su Yeon Rhie, Jae Yong Park, Tae-Seop Shin, Jeong Wook Kim, Beom Jin Kim, Jae Gyu Kim

**Affiliations:** 1 Division of Gastroenterology, Health Promotion Center, Samsung Medical Center, Seoul 06351, Korea; lucidbreeze@naver.com; 2 Department of Internal Medicine, Chung-Ang University College of Medicine, Seoul 06974, Korea; jay0park@naver.com (J.Y.P.); ekg001@cau.ac.kr (J.W.K.); kimbj@cau.ac.kr (B.J.K.); 3 MD Healthcare Inc., Seoul 03923, Korea; tsshin@postech.ac.kr

**Keywords:** *Helicobacter pylori*, antibiotic resistance, fluoroquinolone, mutation

## Abstract

The efficacy of fluoroquinolone-based eradication therapy largely depends on the fluoroquinolone resistance of *H. pylori*. The aim of this study was to investigate the changes in the primary resistance rate of *H. pylori* to fluoroquinolone and the mechanism of resistance in Korea. A total of 153 strains and 48 strains of *H. pylori* were isolated at a tertiary hospital in 2005/2006 and 2017/2018, respectively. The minimum inhibitory concentrations (MICs) of fluoroquinolone were determined by the serial 2-fold agar dilution method. DNA sequences in the quinolone resistance-determining regions of *gyrA*/*gyrB* were analyzed in resistant strains. Subsequent natural transformation study was performed to determine the association between gyrase mutation and resistance. The resistance rates increased from 19.0% (29/153) to 43.8% (21/48) both for levofloxacin and moxifloxacin. The MIC values for resistant strains increased from 2–8 µg/mL to 4–16 µg/mL over time. Mutation of *gyrA* was detected in 93.1% (27/29) and 100% (21/21) among the resistant strains in both periods, respectively. A novel Gly-85 mutation of *gyrA* was found and confirmed to be associated with fluoroquinolone resistance. Fluoroquinolone resistance rate of *H. pylori* has markedly increased over time in Korea. The resistance is mostly due to the point mutation of *gyrA*. Fluoroquinolone-containing regimens should be carefully selected in Korea, considering the increasing fluoroquinolone resistance.

## 1. Introduction

*Helicobacter pylori* (*H. pylori*) infection is recognized as the most important cause of chronic gastritis, peptic ulcer and gastric cancer [1]. Eradication of *H. pylori* has a major role in the treatment and prevention of various gastrointestinal diseases. Standard triple therapy comprising a proton pump inhibitor (PPI), amoxicillin, and clarithromycin has been widely used as a popular *H. pylori* eradication regimen over the world, and is also commonly used as a first-line therapy in Korea. Primary resistance to clarithromycin, which is the most important factor in determining the efficacy of standard triple therapy, was generally acceptable in many countries in early 2000s [2,3]. Recently, however, the eradication rate of standard triple therapy has been constantly decreasing in many regions [4]. This situation is mainly due to increasing resistance to main antibiotic agents used for *H. pylori* eradication, which is partly caused by widespread use of these antibiotics [5]. Therefore, many investigators have searched for alternative eradication regimens in order to achieve successful eradication.

Fluoroquinolone-containing therapy is recommended as one of the rescue therapies after failure of first- or second-line eradication therapies [6]. However, an important caveat of the fluoroquinolone-containing therapy is that the efficacy is markedly diminished in the presence of fluoroquinolone resistance [7,8]. Resistance to fluoroquinolones tends to be easily acquired, and the resistance rate is relatively high in countries where these drugs are widely used [9,10]. The rate of fluoroquinolone resistance, is now increasing over 20% in several countries, where the rate is even higher than 50% in some areas [11,12]. In this situation, when considering fluoroquinolone-containing therapy after other conventional eradication therapies fail, one should take into account the regional resistance rates of *H. pylori* to fluoroquinolone. Moreover, the knowledge of resistance mechanism might be also important in the investigation of changing fluoroquinolone resistance. The aim of this study was to investigate the change in the primary resistance rate to fluoroquinolones and to examine the mechanism of resistance in *H. pylori* isolates from Korean patients over 14 years.

## 2. Results

### 2.1. Patient Demographics and H. pylori Strains

*H. pylori* strains were isolated from 143 patients and 48 patients who underwent upper gastrointestinal endoscopic examination at a single tertiary hospital in 2005/2006 and 2017/2018, respectively. The baseline characteristics of these subjects are shown in Table 1. The mean age of the subjects was 48.6 (19–77) years in 2005/2006, and 61.9 (35–78) years in 2017/2018. The most common endoscopic diagnosis was gastritis in both periods.

*H. pylori* strains were successfully cultured in both the antrum and body from 37/143 patients (25.9%) in 2005/2006, and from 35/48 patients (72.9%) in 2017/2018. Subsequent RAPD fingerprinting was performed in these patients to confirm whether they were different strains, which revealed that 10/37 (27.0%) patients and 0/35 (0%) patients had different *H. pylori* strains in a single host at each time period, respectively. At last, 153 strains and 48 *H. pylori* strains were analyzed at each time period.

### 2.2. Prevalence of Fluoroquinolone Resistance and Distribution of Minimum Inhibitory Concentrations (MICs)

**Figure 1:**
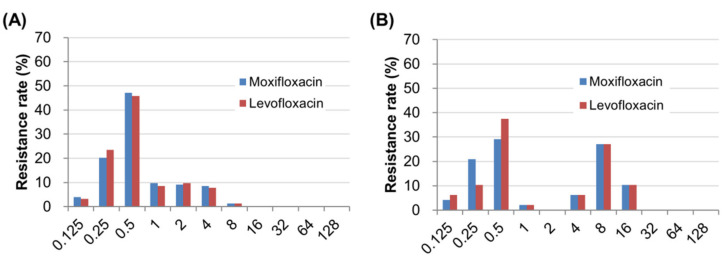
Distributions of MIC values for fluoroquinolones. MIC values for fluoroquinolones of (**A**) 153 strains (2005/2006) and (**B**) 48 strains (2017/2018) are visualized. MICs were determined by the agar dilution method. The MICs values for resistant strains ranged from 2–8 µg/mL and from 4–16 µg/mL at each period. MIC: minimum inhibitory concentration.

The resistance rates for levofloxacin and moxifloxacin were equally 19.0% (29/153) in 2005/2006, which significantly increased up to 43.8% (21/48) for both antibiotics in 2017/2018 (Table 2). In both periods, the resistance to levofloxacin was always accompanied by moxifloxacin resistance, and vice versa, suggesting cross-resistance among fluoroquinolones. The distributions of MIC values for fluoroquinolones were 0.125–8 µg/mL and 0.125–16 µg/mL at each time period (Figure 1). The distributions of MIC values of resistant strains only were 2–8 µg/mL and 4–16 µg/mL at each time period. The mean MIC value of resistant strains significantly increased from 3.31 ± 1.63 µg/mL to 9.33 ± 4.10 for levofloxacin (*p* < 0.001), and from 3.24 ± 1.64 µg/mL to 9.33 ± 4.10 for moxifloxacin (*p* < 0.001), over time.

### 2.3. The Mutation Analyses of DNA Gyrase Genes and the Association with MIC Levels for Fluoroquinolones

The 29 resistant strains obtained in 2005/2006 were analyzed for detection of point mutations in the quinolone resistance determining region (QRDR) of the DNA gyrase genes (Table S1). Among them, 15 strains showed mutations at Asn-87 (51.7%) and 12 strains showed mutations at Asp-91 (41.4%) in *gyrA.* As for *gyrB* mutation, only three strains showed mutations at Asp-495 (10.3%), all of which also had synchronous mutation at Asn-87 in *gyrA*. No mutation was detected in two strains (6.9%) in either *gyrA* or *gyrB*. Among 21 resistant strains from 2017/2018, mutation of *gyrA* was detected at Asn-87 in 62.0% (13/21), at Asp-91 in 23.8% (5/21), at Ala-88 in 4.8% (1/21), simultaneously at Asn-87 and Ala-88 in 4.8% (1/21), and at Gly-85 in 4.8% (1/21) (Table S2). There was no strain with mutation of *gyrB* in this period, unlike in 2005/2006. The Gly85Cys mutation of *gyrA* is a novel mutation that has never been reported before.

There was no specific association between mutation pattern of DNA gyrase and MIC values of fluoroquinolone resistant strains. Point mutations in both the *gyrA* and *gyrB* did not make significant difference in MIC values compared to single mutation in *gyrA*, either (Table 3).

### 2.4. Transformation of H. pylori and Confirmation of Gyrase Mutations Related to Fluoroquinolone Resistance

To determine whether *gyrB* mutation or the novel *gyrA* mutation is associated with fluoroquinolone resistance, natural transformation was performed with the polymerase chain reaction (PCR)-amplified *gyrA* or *gyrB* of resistance strains. Table 4 shows the results of the transformation analysis in 2005/2006. The *H. pylori* ATCC 43504 was used as a negative control and the frequency of transformants was 2.65 × 10^−9^. Transformants resistant to fluoroquinolone were obtained at a frequency of 4.90 × 10^−6^, using a strain with *gyrA* mutation as a positive control. Transformants resistant to fluoroquinolone were obtained at a frequency of 3.25 × 10^−9^, 3.15 × 10^−9^, and 2.20 × 10^−9^ from three strains with *gyrB* mutation, respectively, which was not significantly different from the negative control. Table 5 shows the results of the transformation analysis in 2017/2018. We observed that the MICs of recipient cells (ATCC 43504) transformed by genomic DNAs from the resistant strain with Gly85Cys mutation, were changed from 0.5 to 2 µg/mL for levofloxacin, and from 0.25 to 2 µg/mL for moxifloxacin. Subsequent DNA sequencing of the QRDR of *gyrA* in the recipient cells with acquired resistance revealed mutation from glycine to cysteine at position 85. On the contrary, the same transformation analysis and MIC test followed by sequencing analysis using the resistant strains with Ala88Val mutation showed that there was no change in the MIC value in the recipient strains with Ala88Val mutation.

## 3. Discussion

This study was designed to investigate the changes in the prevalence of fluoroquinolone-resistance of *H. pylori* and the resistance mechanism in Korea over a 14-year period. The primary resistance against fluoroquinolone has increased from 19.0 to 43.8% compared with 14 years ago. Subsequent mutation analyses of DNA gyrase to explore the resistance mechanisms revealed that the resistance was mainly due to the mutation of *gyrA* in both periods.

Increasing resistance to the main antibiotics used for *H. pylori* eradication is a major cause of increasing eradication failure, leading to a significant clinical problem. In Korea, the standard triple therapy has been the most widely used as the 1st-line treatment, and the bismuth containing quadruple therapy is commonly accepted as a 2nd-line therapeutic option for *H. pylori* eradication. However, the efficacy of the standard triple therapy has now decreased to an unacceptable level in Korea, just as many other countries [4,13,14]. In the current situation, various alternative eradication regimens have been proposed.

Efforts have been made to find alternative regimens which could replace conventional therapies. Fluoroquinolone-containing eradication therapy has shown favorable outcomes in several countries, and is regarded as one of the useful alternatives. This regimen is also recommended as a secondary therapeutic option after failure of 1st-line eradication therapy [6,15]. The efficacy of levofloxacin-based triple therapy as first line treatment was reported as 52.5–65.3%, while the efficacy as third line treatment was 71.6% in Korea [16,17,18]. According to this result, levofloxacin-based regimen seems to be a possible option for rescue therapy. However, the clinicians should mind that the success rate of this regimen largely depends on the fluoroquinolone resistance. In previous studies, the efficacy of levofloxacin-based triple therapy as a rescue regimen was 89.8–92.9% for susceptible strains, compared to 12.5–55.6% for resistant strains [7,8]. The efficacy is also variable in different geographic areas worldwide [19]. This might be due to the variable prevalence of resistant strains in different countries, which makes it very important to know the fluoroquinolone resistance profile of *H. pylori* in the community level.

The increasing trend of fluoroquinolone resistance of *H. pylori* is evident in many countries, which can undermine the efficacy of fluoroquinolone-containing regimens. A recent antibiotic resistance mapping study from Korea has shown that the resistant rate of *H. pylori* to ciprofloxacin and levofloxacin has now increased up to 37.0% [20]. The result from our study was consistent with this trend of increasing fluoroquinolone-resistance, showing the resistance rate of 43.8% in 2017/2018. The overall increased use of fluoroquinolones for various infections, such as urinary or respiratory tract infections, might have contributed to the increasing resistance of *H. pylori* [21]. Considering this trend, the alternative regimens including fluoroquinolone should be carefully chosen in Korea.

In this study, all *H. pylori* strains resistant to levofloxacin were also resistant to moxifloxacin, suggesting cross-resistance between fluoroquinolones. This finding is consistent with previous literature, although only few studies have been conducted on this subject [22,23]. The main mechanism of fluoroquinolone resistance in bacteria is to disrupt DNA replication by interfering with DNA gyrase or topoisomerase activities [22]. In fact, *H. pylori* do have neither *parC* nor *parE* genes, which encode topoisomerase IV, and a drug efflux system is considered not to have a major role in showing the resistance [24]. Thus, DNA gyrase has been considered responsible for fluoroquinolone resistance in *H. pylori*. Fluoroquinolone resistance is mainly exerted by affecting the subunit A of the DNA gyrase of *H*. *pylori*, caused by point mutations in the QRDR of the *gyrA* gene [25]. Previous reports have repeatedly shown that the point mutations frequently involve amino acid substitutions at amino acid 87 (Asn87Lys) and amino acid 91 (Asp91Gly, Asp91Asn, and Asp91Tyr) in *gyrA* [22,23,26,27]. Other *gyrA* mutations of Ala88Val, and a double substitution of Asp91Asn and Ala97Val also have been reported [25]. In the present study, more than 90% of resistant strains showed mutation of *gyrA* at either Asn-87 or Asp-91 in both periods. Interestingly, we discovered a novel Gly85Cys mutation of *gyrA*, which had never been previously reported. Subsequent transformation study has revealed that this new mutation in the QRDR of *gyrA* is associated with fluoroquinolone resistance. The Gly85Cys mutation of *gyrA*, discovered only in 2017/2018 period, could mean that there might be some new mechanisms of fluoroquinolone resistance in *H. pylori*, in the era of increasing resistance to fluoroquinolones. On the other hand, only 10.3% of the resistant strains showed *gyrB* mutation in 2005/2006, which were all accompanied by synchronous mutation of *gyrA*. Subsequent transformation experiment revealed that the *gyrB* mutation was not associated with fluoroquinolone resistance. This result was in accordance with previous studies showing that mutation of *gyrB* does not seem to play an important role, if any, in presenting the fluoroquinolone resistance in *H. pylori* [23,27].

The MIC value of resistant strains has significantly increased compared to 14 years ago. However, we failed to show any meaningful correlation between the type of gyrase mutations and MIC values. This finding is consistent with those from previous studies conducted in Asia, which showed no significant association between *gyrA* mutation patterns and the MIC values of fluoroquinolone resistant *H. pylori* strains [28]. In our study, neither double mutations of *gyrA* and *gyrB* have shown any impact on MIC values when compared to single mutation of *gyrA*.

We intended to investigate the primary fluoroquinolone resistance of *H. pylori*. Although we could not investigate whether the subjects had ever taken fluoroquinolone in their lifetime, we have excluded patients with previous history of *H. pylori* eradication therapy, including fluoroquinolone-containing eradication therapy. Knowing the current fluoroquinolone resistance of *H. pylori* and the underlying mechanism is crucial in establishing a strategy for rescue therapies after eradication failure. This can also attribute to deciding whether to start the fluoroquinolone-based treatment empirically or after susceptibility tests. The knowledge of resistance mechanism could be also crucial for searching new antimicrobial agents against multiresistant *H. pylori* strains [29,30].

There are some limitations in this study. First, due to the lack of clinical follow-up data, we did not investigate the eradication results of the study subjects. Therefore, the relationship between the antibiotic resistance profile and eradication results could not be analyzed. This should be further investigated in a well-designed prospective randomized study. Second, this was a single center study conducted in a tertiary hospital, which might have caused some selection bias. Caution is needed when interpreting the result, and applying this result in clinical practice. Nevertheless, this study has strength of being a long-term follow-up study over 10 years which have shown a clear trend of increasing fluoroquinolone resistance. Third, the transformation study was performed with different methods during each period. Even though, as the purpose of transformation study was to find out the underlying mechanism of fluoroquinolone resistance, the usage of difference methods itself has little effect on the interpretation of the results.

In conclusion, the prevalence of fluoroquinolone resistance of *H. pylori* has greatly increased over 14 years in Korea. The resistance is mostly due to the point mutation of *gyrA* gene, while *gyrB* mutation is not associated with resistance. Discovery of a novel mutation in *gyrA* suggests the possibility of emergence of new fluoroquinolone-resistance mechanisms. Fluoroquinolone-containing eradication regimens should be carefully selected for *H. pylori* eradication, considering the high prevalence of fluoroquinolone resistance, which is steadily increasing in Korea.

## 4. Materials and Methods

### 4.1. Study Population

Subjects aged over 19 years old requiring upper endoscopic examination and *H. pylori* test were prospectively enrolled at a single tertiary hospital in Seoul, Korea in 2005/2006 and 2017/2018, respectively. The enrolled patients of the two periods were different subjects. Patients were excluded if they had taken antibiotics within four weeks or had a prior history of *H. pylori* eradication or gastrectomy. All the *H. pylori* strains used in this study were either clinical isolates previously obtained from patients for clinical purposes or *H. pylori* strains obtained from the subjects who were enrolled after informed consent was documented. This study was approved by the Institutional Review Board of Chung-Ang University Hospital (1610-008-259) and conducted in accordance with the Declaration of Helsinki.

### 4.2. H. pylori Culture and Isolation

Two biopsy samples were achieved from each patient from the antrum and the body of the stomach. The *H. pylori* isolates were cultured at 37 °C on Brucella agar plates (Becton Dickinson, Franklin Lakes, NJ, USA) containing 5% defibrinated sheep blood (Hanil Komed, Seongnam, Korea) under microaerobic conditions (5% O_2_, 10% CO_2_, 85% N_2_) for 4 days. Organisms were identified as *H. pylori* by colony morphology, rapid urease test, *H. pylori*-selective media (Oxoid™ SR 147 supplement (Thermo Fisher Scientific, Waltham, MA) and 5% defibrinated sheep blood), and PCR to detect ureA. All stock cultures were stored at −70 °C in Brucella broth supplemented with 15% glycerol (Sigma Chemical Co., St Louis, MO, USA) and 10% fetal bovine serum (Gibco, Grand Island, NY, USA). These preparations were thawed and subcultured for further experiments.

### 4.3. PCR-Based Randomly Amplified Polymorphic DNA (RAPD) Fingerprinting of H. pylori

When *H. pylori* strains were successfully cultured from both the antrum and body in some patients, Randomly Amplified Polymorphic DNA (RAPD) fingerprinting was performed to confirm whether they were different strains. PCR-based RAPD fingerprinting was performed using the method as previously described [31,32]. The primers (Cosmogenetech, Seoul, Korea) used were 5’-AACGCGCAAC-3’ (3881) and 5’-AAGAGCCCGT-3’ (3880). The Ex Taq™ DNA polymerase (Takara Bio, Otsu, Shiga, Japan) was used for PCR amplification, which was performed in a volume of 50 μL, containing 5 μL 10 × Ex Taq™ buffer including MgCl_2_, 4 μL dNTP mixture (2.5 mM each; Sigma Chemical Co., St Louis, MO, USA), 10 pmole of each primer, and 1.25U Ex Taq™ DNA polymerase, following previously described methods [31,32]. A GeneAmp^®^ PCR system 2700 (Applied Biosystems, Foster City, CA, USA) was used for amplification. The PCR profile consisted of 4 cycles of 5 min of denaturation at 94 °C, 5 min of annealing at 36 °C, and 5 min of extension at 72 °C; 30 cycles of 1 min of denaturation at 94 °C, 1 min of annealing at 36 °C, and 2 min of extension at 72 °C; and then 10 min of extension at 72 °C. After PCR-based RAPD fingerprinting, the products were electrophoresed in 1.0% agarose gel (Sigma Chemical Co., St Louis, MO, USA) and photographed under UV light.

### 4.4. Determination of MICs of Fluoroquinolones

The MIC values of the *H. pylori* isolates to levofloxacin (Sigma Chemical Co., St Louis, MO, USA) and moxifloxacin (Bayer AG Pharmaceuticals, Leverkusen, Germany) were examined using the serial two-fold agar dilution method as described previously [33,34]. Briefly, bacteria were subcultured on Mueller-Hinton agar supplemented with 5% defibrinated sheep blood for 48 h. The bacterial suspension, adjusted to MacFaland No. 2 (6 × 10^8^ colony-forming units/mL), was inoculated directly onto each antibiotic-containing agar dilution plate. After 72 h of incubation, the MIC of each antibiotic was determined. Quality control was performed using *H. pylori* ATCC 43504. The resistance breakpoints for levofloxacin and moxifloxacin were defined as >1.0 µg/mL according to the European Committee on Antimicrobial Susceptibility Testing (EUCAST) standards. The above procedure was repeated nine times to determine the MICs of fluoroquinolones.

### 4.5. PCR Amplification and Nucleotide Sequencing

The extraction of *H. pylori* genomic DNA was performed as reported previously [35]. To detect genetic mutation of the QRDRs of the *gyrA* (from codon 38 to 154) and the *gyrB* (from codon 392 to 500) from the resistant strains, the following primers (Cosmogenetech, Seoul, Korea) were used for the PCR; *gyrA* forward, 5’-TTTAGCTTATTCAATGAGCGT-3’ *gyrA* reverse, 5’-GCAGACGGCTTGGTAGAATA-3’ *gyrB* forward, 5’-YGCAAAAGCCAGAGAAGCCA-3’ *gyrB* reverse, 5’-ACATGCCCTTGTTCAATCAGC-3’ [23]. The PCR profile consisted of 40 cycles of 1 min of denaturation at 94 °C, 1 min of annealing at 57 °C, and 1 min of extension at 72 °C [22]. Sequencing was performed with the two strands of the nonrestricted amplicons by use of an ABI PRISM 377 DNA sequencer (Applied Biosystems, Foster City, CA, USA). The sequences were compared with the published sequence of the *H. pylori gyrA* gene (GeneBank accession no. L29481) and *gyrB* gene (GeneBank accession no. NC000915).

### 4.6. Natural Transformation of H. pylori

For the strains obtained in 2005/2006, transformation of *H. pylori* was accomplished by using a modified version of the method previously described [29]. A clinical isolate-*H. pylori* strain susceptible to levofloxacin and moxifloxacin with MIC of 0.125 µg/mL-was chosen as a recipient for transformation experiment on the basis of previous experiments that revealed that it was readily transformed with DNA. The recipient strains were grown for 2 days on sheep blood agar and then subcultured to a new plate in a 1 cm^2^ area. The plate was incubated overnight. After 24 h, 1 µg of donor genomic DNAs in a volume of 200 µL of BHI medium (Becton Dickinson, Franklin Lakes, NJ, USA) was applied to the cells and the plate was further incubated for another 24 h. The cells were then scraped off the plate into PBS, pH 7.2, diluted, and plated onto sheep blood (Hanil Komed, Seongnam, Korea) agar containing 2 µg/mL of levofloxacin or moxifloxacin to select transformants. After 3 days the colonies were counted, the colonies from each transformation were purified and maintained on medium containing levofloxacin or moxifloxacin, respectively. Transformation frequencies were calculated by dividing the number of transformants by the number of viable cells at the time of plating on selective agar. Antibiotic susceptibility test and DNA sequencing of the QRDR of *gyrB* were performed for five colonies from each transformation.

For the *H. pylori* strains obtained in 2017/2018, patient-derived resistant strains were used as donor cells and *H. pylori* ATCC 43504 as recipient cells. The genomic DNAs from the donor cells were used in the natural transformation to evaluate the quinolone-resistance mechanism of *gyrA* mutations at locations other than Asn-87 or Asp-91. After isolation and subculture of *H. pylori* strains was done in the same manner as above, 1 µg of donor genomic DNAs in a volume of 200 µL of BHI medium were applied to the cells and the plate was further incubated for another 24 h. The bacterial suspension was inoculated directly onto each antibiotic-containing agar dilution plate. After 72 h of incubation, the MIC of each antibiotic was determined and DNA sequencing of the QRDR of *gyrA* was performed to confirm the presence of *gyrA* mutation. Antibiotic susceptibility test and DNA sequencing of the QRDR of *gyrA* were performed for three colonies from each transformation.

### 4.7. Statistical Methods

Pearson’s chi square test or Fisher’s exact test was used to compare categorical variables, and Student’s t-test or Mann–Whitney U test was used to compare continuous variables. One-way analysis of variance (ANOVA) test was performed to determine whether there were any statistically significant differences in the mean values of MIC among the fluoroquinolone-resistant groups with different types of DNA gyrase mutation. *p* value < 0.05 was considered statistically significant. All statistical analyses were performed using SPSS version 19 (SPSS Inc., Chicago, IL, USA).

## Figures and Tables

**Table 1:** The baseline characteristics of patients.

Characteristics	2005/2006	2017/2018	*p*-Value
Number of patients (strains)	143 (153)	48 (48)	-
Mean age ± SD (years)	48.6 ± 14.6	61.9 ± 8.7	<0.001
Sex (M/F)	87/56 (60.8/39.2%)	33/15 (68.8/31.2%)	0.326
Endoscopic diagnosis			<0.001
Peptic ulcer disease	65 (45.5%)	15 (31.3%)	
Gastric cancer	3 (2.1%)	11 (22.9%)	
Gastric adenoma	0 (0.0%)	4 (8.3%)	
MALT lymphoma	0 (0.0%)	1 (2.1%)	
Gastritis	75 (52.4%)	17 (35.4%)	

Values are number (%) or mean ± SD unless stated otherwise. SD, standard deviation; MALT, mucosa-associated lymphoid tissue.

**Table 2:** Prevalence of fluoroquinolones resistance among *H. pylori* isolates in 2005/2006 and 2017/2018.

	Prevalence of Resistant Strains (Resistant Strains/Total Strains, %)		
	2005/2006	2017/2018	*p*-Value
Levofloxacin	19.0% (29/153)	43.8% (21/48)	<0.001
Moxifloxacin	19.0% (29/153)	43.8% (21/48)	<0.001

Resistant cut-off values were defined as >1.0 µg/mL for levofloxacin and moxifloxacin, respectively.

**Table 3:** Types of mutations in the QRDR of *gyrA/B* and the MIC values of fluoroquinolone resistant strains.

Group	Substitution in *gyrA*	Substitution in *gyrB*	Number of Patients	MIC (µg/mL) Value			
				LVX		MOX	
				Mean	*p*-Value *	Mean	*p*-Value *
2005/2006 (n = 29)	**Asn-87→Lys**	**Asp-495→His**	3 (10.3%)	4	0.771	4	0.823
	Asn-87→Lys	ND	12 (41.4%)	3.5		3.3	
	Asp-91→Gly	ND	12 (41.4%)	3		3	
	ND	ND	2 (6.9%)	3		3	
2017/2018 (n = 21)	Asn-87→Lys	ND	13 (62.0%)	10.5	0.652	10.5	0.652
	Asp-91→Asn or Gly or Tyr	ND	5 (23.8%)	7.2		7.2	
	Ala-88→Val	ND	1 (4.8%)	8		8	
	Asn-87→Lys& Ala-88→Val	ND	1 (4.8%)	8		8	
	Gly-85→Cys	ND	1 (4.8%)	8		8	

QDRD: Quinolone resistance determining region; MIC: minimum inhibitory concentration; LVX: levofloxacin; MOX: moxifloxacin; ND: not detected. * Statistical significance was tested by one-way analysis of variance among groups.

**Table 4:** Transformation of *H. pylori* Using PCR-amplified *gyrB* DNAs (2005/2006).

Isolated Strains	Amplified PCR Product Used in Transformation	Mutation in Transformed DNA	Transformation Frequency (MIC > 1 µg/mL)		Result
			First	Second	
Negative control Positive control QA2 QA1 QA4 QA12	*gyrB* *gyrA* *gyrB* *gyrB* *gyrB* *gyrB*	None Asn87→Lys None Asp495→His Asp495→His Asp495→His	2.8 × 10^−9^ 5.7 × 10^−6^ 3.0 × 10^−9^ 3.4 × 10^−9^ 2.9 × 10^−9^ 2.5 × 10^−9^	2.5 × 10^−9^ 4.1 × 10^−6^ 4.9 × 10^−9^ 3.1 × 10^−9^ 3.4 × 10^−9^ 1.9 × 10^−9^	2.65 × 10^−9^ 4.9 × 10^−6^ 3.95 × 10^−9^ 3.25 × 10^−9^ 3.15 × 10^−9^ 2.2 × 10^−9^

Negative control: *H. pylori* ATCC 43504; Positive control: PCR-amplified *gyrA* DNA of clinical strain QA2; MIC: minimum inhibitory concentration.

**Table 5:** Transformation of *H. pylori* Using gDNA isolated from resistant strains (2017/2018).

Donor Strain	MIC of Donor Strain (μg/mL)		*gyrA* Mutation of Donor Strain	MIC of Transformed Strains (μg/mL)		*gyrA* Mutation Detected in Transformed Strains
	LVX	MOX		LVX	MOX	
QB1	8	8	Gly-85→Cys	0.5→2	0.25→2	Gly-85→Cys
QB16	8	8	Ala-88→Val	0.5→0.5	0.25→0.25	Ala-88→Val

Donor strain: clinical *H. Pylori* strains from patients; Recipient strain: *H. pylori* ATCC 43504; MIC: minimum inhibitory concentration.

## Supplementary Materials

The following are available online at https://www.mdpi.com/2079-6382/9/6/287/s1, Table S1: Minimal inhibitory concentration (MIC) values and types of mutations in the QRDR of *GyrA/B* of 29 fluoroquinolones-resistant strains in 2005/2006; Table S2: MIC values and types of mutations in the QRDR of *GyrA/B* of 21 fluoroquinolones-resistant strains in 2017/2018.
