# Supplementary material for: Discovery of a Novel Mutation in DNA Gyrase and Changes in the Fluoroquinolone Resistance of Helicobacter pylori over a 14-Year Period: A Single Center Study in Korea
Source: Antibiotics (Basel). 2020 May 27;9(6):287. doi: 10.3390/antibiotics9060287 (PMC7345123; doi:10.3390/antibiotics9060287)
Supplement: Supplementary file 1 [file antibiotics-09-00287-s001.pdf]

antibiotics
MDPI

Table S1

MIC Values and types of mutations in the QRDR of GyrA/B of 29 fluoroquinolones-resistant strains in 2005-2006

Strains	MIC (μg/mL)		Substitution in *gyrA*	Substitution in *gyrB*
	LVX	MOX		
QA1	4	4	Asn-87→Lys	Asp-495→His
QA2	8	8	Asn-87→Lys	ND
QA3	4	4	Asn-87→Lys	ND
QA4	4	4	Asn-87→Lys	Asp-495→His
QA5	2	2	Asn-87→Lys	ND
QA6	4	2	Asn-87→Lys	ND
QA7	2	2	Asn-87→Lys	ND
QA8	2	2	Asn-87→Lys	ND
QA9	2	2	Asn-87→Lys	ND
QA10	4	4	Asn-87→Lys	ND
QA11	2	2	Asn-87→Lys	ND
QA12	4	4	Asn-87→Lys	Asp-495→His
QA13	4	4	Asn-87→Lys	ND
QA14	4	4	Asn-87→Lys	ND
QA15	4	4	Asn-87→Lys	ND
QA16	4	4	Asp-91→Gly	ND
QA17	2	2	Asp-91→Gly	ND
QA18	2	4	Asp-91→Gly	ND
QA19	2	2	Asp-91→Gly	ND
QA20	2	2	Asp-91→Gly	ND
QA21	4	2	Asp-91→Gly	ND
QA22	8	8	Asp-91→Gly	ND
QA23	2	2	Asp-91→Gly	ND
QA24	4	4	Asp-91→Gly	ND
QA25	2	2	Asp-91→Gly	ND
QA26	2	2	Asp-91→Gly	ND
QA27	2	2	Asp-91→Gly	ND
QA28	2	2	ND	ND
QA29	4	4	ND	ND

MIC, minimum inhibitory concentration; QDRD, Quinolone resistance determining region; LVX, levofloxacin; MOX, moxifloxacin; ND, not determined; Asn, Asparagine; Lys, Lysine; Asp, Aspartate; His, Histidine; Gly, Glycine.

Table S2

MIC Values and types of mutations in the QRDR of *GyrA/B* of 21 fluoroquinolones-resistant strains in 2017-2018

**Strains**	**MIC (μg/mL)**		**Substitution in *gyrA***	**Substitution in *gyrB***
	**LVX**	**MOX**		
QB1	8	8	Gly-85→Cys	ND
QB2	8	8	Asn-87→Lys	ND
QB3	4	4	Asn-87→Lys	ND
QB4	16	16	Asn-87→Lys	ND
QB5	8	8	Asn-87→Lys	ND
QB6	16	16	Asn-87→Lys	ND
QB7	8	8	Asn-87→Lys	ND
QB8	4	4	Asn-87→Lys	ND
QB9	16	16	Asn-87→Lys	ND
QB10	16	16	Asn-87→Lys	ND
QB11	8	8	Asn-87→Lys	ND
QB12	16	16	Asn-87→Lys	ND
QB13	8	8	Asn-87→Lys	ND
QB14	8	8	Asn-87→Lys	ND
QB15	8	8	Asn-87→Lys<br>Ala-88→Val	ND
QB16	8	8	Ala-88→Val	ND
QB17	8	8	Asp-91→Asn	ND
QB18	4	4	Asp-91→Asn	ND
QB19	8	8	Asp-91→Asn	ND
QB20	8	8	Asp-91→Gly	ND
QB21	8	8	Asp-91→Tyr	ND

MIC, minimum inhibitory concentration; QDRD, Quinolone resistance determining region; LVX, levofloxacin; MOX, moxifloxacin; ND, not determined; Gly, Glycine; Cys, Cysteine; Asn, Asparagine; Lys, Lysine; Ala, Alanine; Val, Valine; Asp, Aspartate; Tyr, Tyrosine.
